# Psychological First Aid Well-Being Support Rounds for Frontline Healthcare Workers During COVID-19

**DOI:** 10.3389/fpsyt.2021.669009

**Published:** 2021-05-28

**Authors:** Mansoor Malik, Jessica Peirce, Michael Van Wert, Cynthia Wood, Haroon Burhanullah, Karen Swartz

**Affiliations:** ^1^Department of Psychiatry, Johns Hopkins University, Baltimore, MD, United States; ^2^Johns Hopkins Bayview Medical Center, Baltimore, MD, United States

**Keywords:** psychological first aid, front line healthcare workers, COVID-19, psychological stress, staff support

## Abstract

**Background and Objective:** Frontline healthcare workers face unprecedented stress from the current SARS COV-2 (COVID-19) pandemic. Hospital systems need to develop support programs to help frontline staff deal with this stress. The purpose of this article is to describe a support program for front line healthcare workers.

**Methods:** In this community case report, we describe a well-being support rounding program that was developed to deliver Psychological First Aid (PFA) to frontline healthcare workers in a large urban medical center to maintain their sense of psychological well-being and self-efficacy. A team of clinicians from the department of psychiatry, who were trained on the Johns Hopkins RAPID model (Reflective Listening, Assessment, Prioritization, Intervention, and Disposition) to provide PFA, were deployed throughout the hospital. These clinicians carried out daily well-being rounds from April to June during the peak of the pandemic.

**Results:** Approximately 20% of the frontline staff members were going through an acute crisis and benefited from PFA. Anxiety, anger, exhaustion, economic worry, job insecurity, dehumanized interactions with patients due to Personal Protective Equipment (PPE), and stress of taking care of sick and dying patients without their families present, were the main themes identified by the staff. The deployed team used active listening, mindfulness, validation, reframing and other cognitive interventions to support staff.

**Conclusions:** Our experience suggests that frontline staff are willing to engage with in-person, on-site support programs. Fostering resilience and self-efficacy through PFA is a useful model to provide emotional support to frontline healthcare workers during health crises.

## Introduction

Previous experience has shown that healthcare workers (HCW) are unlikely to actively seek out emotional help. During the 2003 SARS outbreak, HCW did not seek out formal mental health support, but did seem to benefit from peer support when it was available (1). More recently in a study in China during COVID-19, staff showed a marked reluctance to participate, as well as refusal by some staff to accept help despite showing irritability, unwillingness to rest, and other signs of psychological distress. However, when counselors were available in the rest areas of HCW's, medical staff spontaneously voiced their experiences as a form of stress relief (2). Therefore, it is important to devise peer support programs during COVID-19 that have direct outreach to vulnerable staff. Here we describe the process of creating and implementing a peer support intervention to provide psychological first-aid to frontline healthcare workers at Johns Hopkins Bayview Medical Center (BMC) during COVID-19.

## Methods

BMC is one of five regional hospital campuses in the Johns Hopkins Medicine network. BMC is a tertiary care center with over 3,000 employees and 710 physicians. Prior to COVID-19, there was no onsite employee assistance program. We sought to design and implement a peer support program with the following goals: (1) Support HCW in maintaining their sense of psychological well-being and self-efficacy so they could continue to do their work during the pandemic and to reduce the likelihood of posttraumatic stress reactions or burnout, (2) to identify and provide interventional support to at-risk individuals with lower resilience, inadequate or inappropriate coping, or other signs of acute stress, (3) provide upstream referrals and to actively facilitate connection with psychiatric services or resources for those individuals who experienced dysfunction due to acute stress, and (4) identify broad stress-related themes, convey wellness-related concerns to the hospital command center, and coordinate efforts with pastoral services and other support initiatives.

Twelve members from Department of Psychiatry and Behavioral Sciences volunteered to participate in the well-being support rounds: two psychiatrists, one psychologist, two nurses, and seven mental health therapists. They were trained on The Johns Hopkins model of psychological first aid (RAPID-PFA) (Reflective listening, Assessment, Prioritization, Intervention and Disposition) (3) through a 6-h online course. Unlike traditional psychiatric interventions, Psychological First Aid does not pathologize people who are stressed by extraordinary events. Rather, it assumes that those who are stressed are competent and are able to determine whether or not they wish or need assistance. Fostering self-efficacy, resilience, and optimism through PFA has been shown to improve coping and efficiency during disasters (4).

Team members rounded on COVID-19 units daily, including weekends. An average round would take about 2 h and 20–25 staff contacts were made in each round. Support rounders would prompt about 5–10 in-depth discussions with staff who wanted to express their concerns, wanted guidance about a specific topic, or just wanted to express their emotions. Support rounders carefully recorded and reported the themes expressed by staff which were discussed in weekly calls with Department of Psychiatry and Behavioral Sciences leaders. Additional vulnerable staff members were identified through discussion with unit supervisors and pastoral services as well as among the members of support group. Vulnerable workers were defined as workers with known risk factors such as younger age, medical issues, or showing signs of psychological distress such as crying and poor coping.

## Results

A total of 1,319 staff were contacted during well-being rounds from March to June (Table 1). Of these, 272 (20.6%) were going through a crisis by their self-report and were provided with PFA. We did not collect formal measures of distress or outcome data as these were not patients or research subjects, and the interventions were performed during a public health emergency. We estimate that 90% of staff members were nurses and support staff, and the rest were attending physicians and residents.

**Table 1:** Well-being support round services offered, April–June 2020.

	**April**	**May**	**June**
# of units visited	112	129	114
# of check-ins with staff	474	460	381
#PFA sessions with staff	88	108	76

Anxiety, anger, and exhaustion were the themes identified early on during the discussions with staff. BMC saw a surge of COVID-19 cases starting in early March. Between March and June, BMC had more than 700 COVID-19 positive admissions, many of them with serious complications. To accommodate this rapid influx of patients, two medical units (MICU and CCU), as well as surgical recovery rooms were converted to COVID-19 units. These units had to quickly be updated with negative pressure biocontainment equipment and donning and doffing stations for PPE were set up. Staff were required to adjust to their new roles and routines overnight. Many nursing and support staff were redeployed to other units, thus breaking up their peer structure. Staff reported feeling exhausted, often with no time to take breaks to eat as it took much longer to do their work due to PPE and other restrictions. Staff felt vulnerable and unappreciated. Many staff members reported dehumanized interactions with patients due to PPE as a major source of their frustration.

In these early days, there was still a lot of uncertainty about the transmission of the novel coronavirus, as well as a significant shortage of PPE and testing. Staff were feeling increasingly anxious during this transition. Many of them were asked to work long shifts in an unfamiliar professional environment while dealing with COVID-19-related stresses at home. Many of them had to arrange childcare for their now out-of-school children and felt worried about taking the infection home. Many were not able to see their elderly parents, while others were staying in the basements of their homes and not having any contact with their families for weeks.

After the initial period of shock, the economic concerns around furloughs and reduced work hours became prominent. Many staff members were worried about job security as some of the non-COVID-19 units were closed. Redeployed staff identified increased stress and anxiety related to necessary adjustment to different units and new roles. As the pandemic increased in intensity, staff had to treat much sicker patients and reported stress of giving bad news to patients without their families to support them. They identified increased stress of caring for dying patients when family could not be present with patients. The concerns expressed by the staff are summarized in Table 2.

**Table 2:** Themes that emerged from speaking with clinical staff, providers, and support staff among COVID-19 and non-COVID-19 units.

**April/May 2020**	**June 2020**
Increased anxiety Anger Feeling underappreciated Worry about own health Worry about taking illness home to family Increased stress of caring for dying patients when family cannot be there with the patient Exhaustion No time to take break to eat Takes longer to do all work Feeling vulnerable Skin irritation re: face mask Stress as not all staff wear PPE correctly	Increased anxiety re: furloughs, layoffs, who will be next Stress of giving bad news to patients without their family there to support them Job security as units closed Unable to work enough hours as units, clinics close, decreased hours Increased stress, anxiety of adjustment to different unit, new role Loss of staff, providers as they are called back to home unit areas as they reopened Sicker patients Decrease in support roles to COVID-19 units (runners, Safety Officers) Worry regarding increase of COVID-19 patients to ED

Support rounders identified vulnerable staff through discussion with supervisors, chaplaincy, or other resources in the unit. Initially, staff members were hesitant to open up to support rounders. As the team showed consistent availability in the units, staff developed a level of trust and frequently approached them. The team frequently rounded at change of shift and weekends when staff were more willing to talk. The team also approached staff in break rooms when they felt at ease and willing to talk freely.

The team used active listening techniques to understand the vital aspects of staff's traumatic experience and establish rapport. Functional assessment was carried out to determine behavioral functioning and ability to carry out clinical duties. Staff members with a high risk of developing further complications were prioritized for further intervention. This entailed teaching stress management techniques and mindfulness. Staff members that continued to show significant levels of distress were referred for psychiatric evaluation.

Psychological interventions used are summarized in Table 3. Some examples of the interventions by the team are as follows.

**Table 3:** List of psychological interventions used during the study.

Fostering contact and engagement
Imparting a sense of safety and comfort
Information gathering: needs and current concerns
Providing information on coping
Cognitive reframing
Self-compassion
Validation
Positivity
Fostering self-efficacy
Providing practical assistance
Linkage with collaborative services
Connection with social supports

Cognitive Reframing: “Recognize the valuable role you and your colleagues play on the front lines of COVID-19. Remind yourself that despite challenges, you are making a difference and taking care of those most in need.”

Self-Compassion: “You are doing the best you can in a difficult situation. Bad things can happen to your patients despite your best efforts. It's not your fault.”

Validation: “There is no right or wrong way to process the COVID-19 experience. It is normal to feel a range of emotions including being overwhelmed, frustrated or angry, worried, anxious, restless, agitated, sad or fatigued. It is not a sign of weakness to feel like that.”

Positivity:” It can be easy to get overwhelmed hearing about the growing number of confirmed cases, shortage of resources and loss of life. Try to find the hopeful stories about communities coming together to recognize front-line workers, like you, for their sacrifice.”

Although we did not collect formal outcome data, as this was not a research study, anecdotal evidence (including the experience of the authors, report from support team members, and feedback received from HCW's, as well as hospital administrators) suggests that the interventions by our team were perceived to be very beneficial. Some of the comments we heard from staff were, “it's thoughtful,” “kind to round on us,” and “it helps to talk.”

## Discussion

Although many institutions have started peer support groups during COVID-19, so far, there is only limited data available for the support of frontline healthcare workers. Viswanathan et al. (5) report on an online peer support group in New York that incorporates some aspects of PFA. Similarly, Francis et al. (6) report on a program to deliver remote PFA in a hospital in Mayasia. Albott et al. (7) report on peer support efforts based on a military battle buddy model for frontline healthcare workers. It is our hope that our experience may provide direction to others who seek to implement their own well-being programs during COVID-19.

## Data Availability Statement

The original contributions presented in the study are included in the article/supplementary material, further inquiries can be directed to the corresponding author.

## Author Contributions

All authors listed have made a substantial, direct and intellectual contribution to the work, and approved it for publication.

## Conflict of Interest

The authors declare that the research was conducted in the absence of any commercial or financial relationships that could be construed as a potential conflict of interest.
